# Supplementary material for: A robophysical model of spacetime dynamics
Source: Sci Rep. 2023 Dec 7;13:21589. doi: 10.1038/s41598-023-46718-4 (PMC10703937; doi:10.1038/s41598-023-46718-4)
Supplement: Supplementary file 2 — Supplementary Information. [file 41598_2023_46718_MOESM2_ESM.pdf]

Scientific Reports Supplementary Information

A robophysical model of spacetime dynamics

Shengkai Li[1], Hussain N. Gynai[2], Steven Tarr[2], Emily Alicea-Muñoz[2], Pablo Laguna[3], Gongjie Li[2], Daniel I. Goldman[2*]

[1] Department of Physics, Princeton University, Princeton, 08544, New Jersey, USA

[2] School of Physics, Georgia Institute of Technology, Atlanta, 30332, Georgia, USA

[3] Center for Gravitational Physics, Department of Physics, University of Texas at Austin, Austin, 78712, Texas, USA

Supplementary movie

This movie first shows the trajectories of a passive marble and an active vehicle in experiments. The passive marble and the active vehicle started with the same initial radius and velocity. Then, it shows the trajectories of a simulated active vehicle for different inverse latus recta and different semi-major-axes.

Raw data

The MATLAB code **plotExperiment.m** in the zip file uses the raw data in the same folder to plot the trajectories, the time evolution of the radius, and the time evolution of the speed for the passive marble and the active vehicle (Fig.2b in the main manuscript). The curves for the passive marble and the active vehicle are shown in blue and red respectively.
